# A scoping review of olfactory interventions for fatigue relief: addressing occupational health hazards

**DOI:** 10.3389/fpubh.2024.1409254

**Published:** 2024-06-28

**Authors:** Xinyue Jiang, Kanesan Muthusamy, Xueliang Fang

**Affiliations:** ^1^Department of Mechanical Engineering, Faculty of Engineering, Technology and Built Environment, UCSI University, Kuala Lumpur, Malaysia; ^2^Department of Electronic Engineering, Yangzhou Technical Vocational College, Yangzhou, China; ^3^College of Traffic Engineering, Yangzhou Polytechnic Institute, Yangzhou, China

**Keywords:** scent, alertness, fatigue, olfactory, scoping review, occupational health hazards

## Abstract

**Background:**

Fatigue poses risks to occupational health and safety, affecting individuals' work efficiency, physical health, and social security, as well as human wellbeing and quality of life. Olfactory interventions, due to their low interference, are considered promising strategies for mitigating fatigue and reducing occupational health hazards.

**Objective:**

The objective of this review is to bridge the current gaps in the literature by conducting a scoping review of olfactory interventions on human alertness. It aims to explore their application in various occupational settings and to provide comprehensive and practical guidance for the practical application of olfactory interventions in mitigating fatigue and reducing occupational risks.

**Methods:**

The literature research was conducted in English using electronic databases such as Web of Science. Keywords related to scent and fatigue and the review followed PRISMA Extension for Scoping Reviews and PICO framework.

**Results:**

28 studies were included in this work. Participant characteristics, fatigue measurement methods, and scent intervention methods, such as types of scents, intervention strategies, and scent presentation systems, are thoroughly investigated and discussed. Additionally, the study places a specific emphasis on the applications and research within the field of scent interventions for fatigue driving. Olfactory interventions have been applied to populations in various occupational fields, demonstrating beneficial effects on both physiological and psychological fatigue.

**Conclusions:**

Olfactory intervention is effective and promising for enhancing alertness and improving the occupational environment. To provide detailed and practical guidance for the actual application of olfactory intervention in fatigue relief and reducing occupational health and safety hazards, further research into the potential mechanisms, applications, and efficacy assessment systems of fatigue-related olfactory interventions is necessary.

## 1 Introduction

Extended work duration is associated with fatigue, which represents a significant occupational safety and health hazard. Fatigue affects both efficient work and physical wellbeing. Research indicates that fatigue can lead to respiratory issues, insomnia, depression, and isolation (1, 2). Additionally, chronic fatigue sufferers may be at risk of suicide (3). Fatigue also gives rise to occupational and societal safety issues, impacting people's overall wellbeing. Reviewing accident reports reveals that ~20% of aviation accidents are related to fatigue (4). Experts suggest that attributing 20%−30%of traffic accidents to driver fatigue is a conservative estimate (5). Surveys among healthcare workers, predominantly nurses, indicate that 19% believe fatigue contributes to the deterioration of patients' conditions (6).

Employing appropriate methods and techniques to alleviate fatigue and reduce occupational risks is crucial for enhancing occupational health and safety. Numerous studies have devised various strategies to prevent and alleviate fatigue, such as music (7, 8), temperature regulation (9, 10), light, and caffeine (11), among others. It is noteworthy that olfactory intervention, as a method to improve fatigue, has gained attention for its minimal interference with regular activities and work, making it a promising approach.

The sense of smell influences alertness and fatigue in humans through mechanisms such as emotional regulation, neural impact, cognitive influence, and physiological responses (12, 13). Emotional regulation refers to how certain odors can modulate one's emotional state, enhancing pleasure, reducing anxiety, and alleviating stress, thereby enhancing alertness and reducing fatigue. Neural impact refers to the interaction between odors and the sensory organs, which transmit signals to the brain and interact with the nervous system. Certain odors can stimulate neural activity and promote efficient neural transmission, thus enhancing alertness and reducing fatigue. Cognitive influence involves the association of specific odors with memories and experiences. When exposed to particular odors, relevant memories and experiences can be evoked, stimulating positive emotions and attention, thereby increasing alertness and reducing fatigue (14). Physiological responses occur when odors affect various physiological indicators in the body, such as heart rate, blood pressure, and brain waves (15, 16). Certain odors can modulate these physiological markers, promoting relaxation and recovery, thus improving alertness and reducing fatigue.

Although numerous studies have investigated the impact of odors on human alertness, there remains a gap in the literature, hindering the practical application of olfactory interventions in mitigating fatigue and reducing occupational risks. To address this gap, a scoping review was conducted to systematically map the research landscape in this area and identify any existing knowledge deficits. This study aims to explore the participant demographics, fatigue measurement methods, and types of intervention odors in current olfactory intervention research, particularly in occupational fields such as fatigue driving. It also discusses the trends in the development of fatigue detection methods and olfactory intervention methods, including scent intervention strategies and intervention strategies. By attempting to fill the gaps in the literature, this research aims to position odor as a novel strategy for preventing occupational health hazards, alleviating the occupational risks associated with fatigue, and protecting and promoting the wellbeing of workers worldwide.

## 2 Research methods

### 2.1 Literature search

This study was carried out in accordance with PRISMA Extension for Scoping Reviews (17). The literature research was conducted between January 5th and February 20th, 2024 on Web of Science Core Collection, MEDLINE, BIOSIS Previews, SciELO Citation Index and Google Scholar. The selected articles were published between 2013 and January 2024 and were composed in the English language. The review also encompassed research in the field of traffic safety and relevant government websites. In addition, reference lists of identified articles were examined, and manual searches were conducted for conference proceedings to further enhance the research.

Conducting electronic database searches using the following keywords: scent, odor, olfaction, fragrance, aroma, flavor, smell, alertness, awareness, sleepy, fatigue and their relevant olfaction synonyms. The following combinations of keywords were employed in advanced searches across accessible databases:

A: TS = alertness AND (TS = fragrance OR TS = scent OR TS = odor OR TS = aroma)
B: (TS = sleepy OR TS = fatigue OR TS = tired OR TS = drowsiness) AND (TS = fragrance OR TS = scent OR TS = odor)
C: (TS = alertness OR TS = awareness OR TS = sleepy OR TS = fatigue) AND (TS = olfaction OR TS = smell)
D: (TS = alertness OR TS = fatigue OR TS = drowsiness) AND (TS = fragrance OR TS = scent OR TS = odor OR TS = aroma OR TS = perfume)

### 2.2 Inclusion and exclusion criteria

This study reviews relevant literature based on the PICO framework. In this research, the PICO elements are as follows:

P - Participants,
I - Intervention or Exposure = Any scent interventions or exposures,
C - Comparison or control group = No intervention or exposure in the study,
O - Outcome = increased alertness.

After removing duplicates, the two authors independently screened the studies based on the review criteria, following a two-step process: (1) screening based on titles and abstracts, and (2) full-text screening to determine the theoretical application, methodology, key findings, and discussion of the studies. In cases where there was any uncertainty regarding the inclusion of an article, the authors discussed and reached a consensus.

### 2.3 Data extraction and summarization

The complete reading of articles allowed us to extract data on: authors, years of publication, study locations, aims, designs, participants' characteristics, measurement methods, intervention strategies and main results.

Two reviewers independently extracted and collated data from each eligible article. Any disagreements were resolved through discussion between the two reviewers.

We summarized the populations, intervention strategies and study designs for each group, along with the measures used and broad findings.

## 3 Results

### 3.1 Article selection

**Figure 1:**
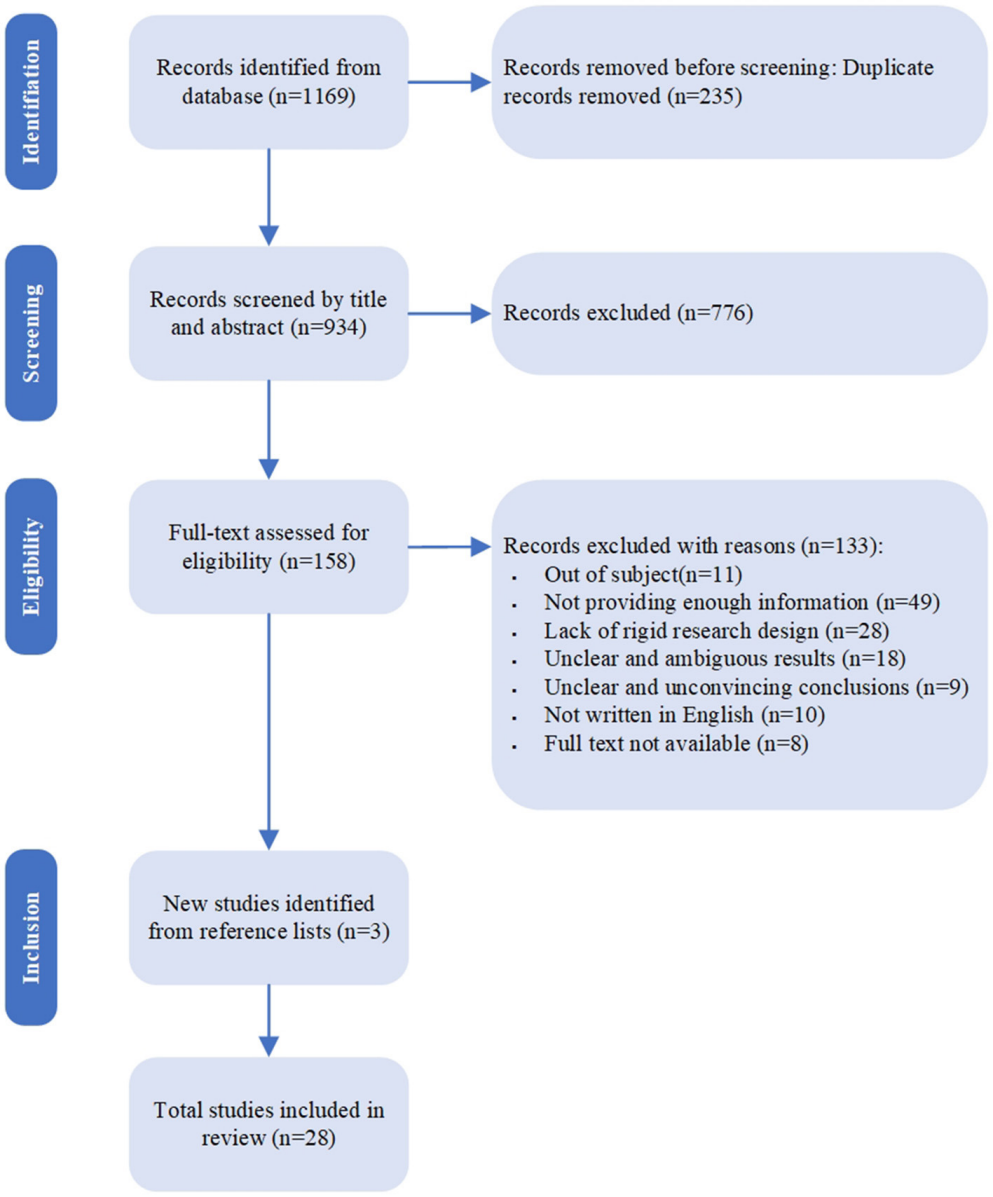
Flow diagram of literature selection.

The flow diagram (Figure 1) presents the results for each step. Before the screening, 235 papers were removed because they were duplicates, leaving 934 articles for later analysis. Based on the title and the abstract, 779 were excluded, with 155 full-text articles to be retrieved and assessed for eligibility. Of these, 133 were excluded for the following reasons: 11 were deemed unrelated to the subject, 49 did not provide sufficient information, 28 lacked a rigorous research design as they did not employ randomized controlled trials (RCTs) or non-randomized controlled trials (nRCTs), 18 used inappropriate measurement methods and failed to clearly present results through physiological, subjective, or behavioral methods, 9 had unconvincing conclusions due to a high level of speculation without empirical support, and 10 were not written in English. We excluded 8 studies because we were unable to retrieve them. The remaining 25 studies were considered eligible for this review. Following further examination of the citation lists in the articles, 3 additional papers were deemed to meet the criteria. In total, 28 articles met the criteria for this review.

### 3.2 Research on scent intervention on fatigue driving

In studies focusing on reducing occupational hazards through olfactory stimulation, one of the most prominent research areas is the impact of olfactory stimulation on fatigue driving. Odors can enhance driver alertness, effectively alleviate driver fatigue, mitigate occupational risks, and reduce the risk of traffic accidents. Detailed information on relevant studies concerning scent interventions for fatigue while driving is presented in Table 1.

**Table 1:** Studies on scent intervention for fatigue driving.

**Reference**	**Year**	**Nation**	**Participants**	**Measurement**	**Intervention/exposure**	**Aim**
Pujiartati et al. (18)	2019	Indonesia	100 participants aged between 17 and 39	Questionnaire		Assesses preferences for three stimulant odors: peppermint, rosemary, and basil essential oil
			12 males	EEG Heart rate KSS	(1) No peppermint odor exposure (2) Peppermint odor exposure during simulation (3) Exposure for 3 consecutive nights before simulation	Examines the impact of prolonged peppermint aroma exposure on simulated driving awareness
Yoshida et al. (19)	2011	Japan	10 licensed drivers in their 20s	Eyeblink frequency	Peppermint, rosemary, eucalyptus and lemon fragrance	Examines the effectiveness of olfactory stimuli in combating driving drowsiness
Hirata (20)	2001	Japan	4 participants	Questionnaire	Peppermint scent	Evaluates scent's role in sustaining alertness and evaluates this in a real vehicle experiment
Funato et al. (21)	2009	Japan	4 males in their 20 s	Blinking Steering characteristics Questionnaire	Peppermint fragrance	Verifies effectiveness of scent projector for reviving driver's alertness and investigates duration of single olfactory emission's stimulation effect.
			18 males in their 20 s		(1) Continuous Scent Presentation (2) Intermittent Scent Presentation	Explores efficient fragrance presentation technique for driver stimulation enhancement
Tang et al. (22)	2021	China	60 participants aged between 22 and 57	Reaction time Time to collision Maximum lateral acceleration Maximum deceleration Overshoot HRV	Auditory beeps Wristband vibrations Peppermint odor	Measures the influence of olfactory stimulation and takeover modality on conditional automated driving takeover performance
Bordegoni et al. (23)	2016	Italy	15 participants	Blood Volume Pulse HRV Questionnaire	Peppermint smell Auditory stimuli	Examines the potential of olfactory stimuli to impact the cognitive aspect of the driver
Jiang et al. (24)	2023	China	11 male subjects aged between 20 and 35	EEG KSS	Peppermint scent	Explores the application of scent as a timely intervention measure for fatigue driving

EEG, Electroencephalograph; KSS, Karolinska Sleepiness Scale; HRV, Heart rate variability.

In terms of scent types, peppermint is considered popular and effective in enhancing driver alertness. A survey of 100 participants on the preference for three scents of peppermint, rosemary, and basil essential oils showed that peppermint was the most favored scent (18). Yoshida et al. found that peppermint, rosemary, eucalyptus and lemon scents can all maintain driver vigilance and prevent drowsy driving, with peppermint being the most effective (19).

Previous studies have reported different scent exposure strategies, including long-term vs. short-term exposure, continuous vs. intermittent exposure and exposure during driving vs. after fatigue. 12 participants underwent a 2-h simulated driving task under three conditions: (a) no peppermint scent, (b) short-term exposure to peppermint scent during the simulated driving task, and (c) long-term exposure to peppermint scent for three consecutive nights before the simulated driving task (18). The results showed that both short-term and long-term exposure to peppermint scent improved driver awareness, with no notable disparity in effectiveness between the two exposure conditions. Hirata conducted an experiment in a truck cab, measuring the characteristics of smell, investigating the time and interval of scent release during driving (20). The study found that the most effective method of releasing scents during driving was to release them for 1 min and then fluctuate the release interval between 5 and 9 min. After driver fatigue, a 30-s interval of peppermint scent ensured 16 min of wakefulness (19). It can be observed that the frequency and duration of scent exposure can affect the effectiveness of enhancing driver alertness.

### 3.3 Participants' characteristics

As shown in Table 2, the studies discussed in this paper cover a variety of participants, including individuals from different occupational backgrounds.

**Table 2:** Studies on Scent Intervention for alertness.

**Reference**	**Year**	**Nation**	**Participants**	**Measurement**	**Intervention/exposure**	**Main results**
Akhule et al. (25)	2021	Iran	97 surgical technologists	MFI	Four times a week for six weeks: G1: Exercise G2: Inhaled Lavender fragrance (2 h) C: No intervention	Exercise and lavender groups exhibited noteworthy decreases in average fatigue scores compared to the control group.
Nasiri et al. (26)	2021	Iran	80 shift-working nurses	KSS Epworth Sleepiness Survey	Applied rosemary oil to gauze in a mask. Wore for 10 min, removed 15 min, repeated 2 h.	Nurses exposed to rosemary aroma intervention exhibited significantly higher alertness compared to the control group.
Mahdavikian et al. (3)	2021	Iran	105 cardiac patients	Fatigue Severity Scale	At 9 PM, 20 min, for 7 nights: G1: 3 drops peppermint oil on a cotton ball on the collar G2: 3 drops lavender oil C: Aromatic distilled water	The lavender and peppermint groups experienced a reduction in average fatigue levels, with statistically significant differences compared to the control group.
Shirzadegan et al. (27)	2020	Iran	80 patients with Acute Myocardial Infarction	STAI MFI	Participants wore an oxygen mask with citrus essential oil or a placebo patch for 20 minutes, repeated for two consecutive days.	In all four measurements after the intervention, the citrus aurantium essential oil was found to reduce anxiety and fatigue compared to before the intervention.
Hur et al. (28)	2019	South Korea	62 prediabetic middle-aged women	Numeric rating scale Objective stress index Fructosamine Verran & Snyder-Halpern Scale	A blend of lavender, geranium, cinnamon, grapefruit, neroli, and Ylang Ylang in a specific ratio was used. The intervention group wore an oil necklace all day and did daily 15–20 min abdominal massages with the oil.	Subjective stress differed significantly after 2 weeks between the groups. Treatment led to significant changes in fructosamine, fatigue, and sleep quality.
Varaei et al. (29)	2020	Iran	96 hemodialysis patients	Rhoten fatigue scale	Three times a week for 8 weeks: G1: Lavender and sweet orange essential oil on a cloth placed on the shirt collar (20 min) G2: Masseuse massaged feet with essential oil (10 min)	After 8 and 16 weeks of study, both groups exhibited significantly lower fatigue levels compared to the control group.
Tianlong and Sim (30)	2019	Korea	12 female adolescent boxers	Lactic acid Creatine phosphokinase Lactate dehydrogenase Cortisol Epinephrine Norepinephrine Adrenocorticotropic hormone levels	Participants engaged in 5 rounds of 4-min boxing sparring (70%-80% max heart rate) before interventions: G1: Static rest (20 min) G2: Massage (20 min) G3: Aromatherapy (rosemary) (20 min) G4: Acupoint acupressure (20 min)	Interventions (static rest, massage, aromatherapy, acupoint acupressure) reduced lactic acid levels significantly. Aromatherapy and acupoint acupressure decreased creatine phosphokinase levels, while massage, aromatherapy, and acupoint acupressure lowered lactate dehydrogenase levels significantly. Aromatherapy decreased cortisol and adrenocorticotropic hormone levels significantly, whereas static rest increased cortisol levels. The aromatherapy group also showed notable reductions in adrenocorticotropic hormone levels, similar to massage and acupressure trends.
Kwon et al. (31)	2020	Korea	20 university students engaged in regular aerobic exercise	In-Depth interview Feeling Scale Felt Arousal Scale	Participants smelled an orange scent during a 30-min treadmill running session.	Aromatherapy improves the exercise experience, reduces fatigue, and enhances recovery feelings.
Anu et al. (32)	2022	India	75 students aged between 18 and 20	Visual Reaction Time HR Perceived Stress Scale questionnaire Memory test	G1: Chew peppermint-flavored gum G2: Chew flavorless gum C: No gum Task: Read a Parkinson's disease topic (30 min), followed by a test and a retest after a month.	Significant increase in short-term memory and HR after intervention. Short-term memory levels among the three groups differed significantly. G1 had significantly higher long-term memory scores after one month compared to other groups.
Lwin et al. (33)	2021	USA	73 undergraduates	Mean Reciprocal Reaction Time Attentional lapses Identification of suspicious cues Eye tracker measures Memory test	G1: Continuous peppermint scent G2: Peppermint scent every 10 minutes C: No scent Task: Monitor surveillance recordings of four screens to identify suspicious events (1 h and 45 min)	Scent improved recall and countered alertness decline, particularly with fatigue. Intermittent emission had a slightly stronger impact on alertness and memory compared to continuous emission.
Asikin et al. (34)	2019	Japan	9 healthy adult female participants	Profile of mood states test EEG	Participate in a 30-min VDT test while inhaling Shiikuwasha (Citrus depressa Hayata) fragrance with limonene and γ-terpinene	Adding a refreshing lemon-like scent during VDT work in the SEO stream slightly improved participants' psychological mood, enhancing vigor and reducing fatigue. EEG recordings during γ-terpinene exposure consistently showed lower beta and theta spectral power at the midline occipital electrode, potentially enhancing focus and reducing anxious thinking during VDT work.
Martial et al. (35)	2023	Belgium	21 healthy young men aged between 21 and 30	Magnetic resonance imaging data Positive and Negative Affect Schedule KSS STAI	G: Lemon scent C: no scent	Inhaled lemon fragrance increased alertness. Functional connectivity analysis showed enhanced thalamus connectivity and reduced connectivity in sensory cortical areas. Graph theory analysis revealed improved integration in olfactory and emotion processing regions and reduced segregation in posterior brain regions during olfaction.
Ohata et al. (36)	2022	Japan	32 healthy participants aged between 20 and 24	Pupillary light reflex Fingertip temperature Near-infrared spectroscopy VAS Multiple mood scale	G1: Inhaled iyokan essential oil G2: Inhaled yuzu essential oil	Inhalation of both essential oils increased miosis rate and fingertip temperature, inducing parasympathetic dominance by suppressing sympathetic nerve activity. Participants felt less fatigued and more refreshed. Yuzu oil raised prefrontal oxyhemoglobin, while iyokan oil decreased it. Task performance notably improved with yuzu oil inhalation.
Pan et al. (37)	2020	Japan	72 participants aged between 20 and 30	POMS The User Experience Questionnaire VAS	G1: Sweet orange scent G2: Peppermint scent C: No scent Task1: View completed blocks and additional blocks Task2:Enjoy blocks within 12 minutes	The sweet orange scent is most preferred, reducing fatigue. Peppermint aroma eased task-induced tension and boosted energy.
Yu et al. (15)	2022	China	40 healthy participants	HR Systolic blood pressure Diastolic blood pressure HRV Salivary alpha-amylase Semantic differential POMS	Hinted group: Read text (3 min) Non-hinted group: Rest (3 min) Taiwania oil odor exposure: 2 min.	Taiwania's scent reduced heart rates, suppressed negative moods (confusion, fatigue, depression), and enhanced affective scores in stimulation, excitement, firmness, distinctiveness, activity, and denseness, but lower pleasantness. Positive cognitive bias reduced anger-hostility feelings and alleviated unpleasantness toward the smell.
Kim et al. (38)	2022	Korea	26 university students	HRV POMS STAI	G: Inhaled Fir essential oil (3 min) C: Inhaled air (3 min)	The improved positive mood of “vigor” and decreased negative moods of “tension-anxiety”, “depression”, “anger-hostility”, “fatigue”, and anxiety levels with fir essential oil inhalation.
Shen et al. (39)	2021	China	20 healthy adults	POMS Japanese Subjective Fatigue Symptom Questionnaire	60 min in wooden structures of DR, HR, and LR.	Females felt warmer and brighter in wooden rooms. Females had 42% higher olfactory sensation than males in a dark wooden room, decreasing after a 50-min adaptation. Females reported more confusion and fatigue, while males reported more vigor in varying conditions.
Koomhin et al. (16)	2020	Thailand	25 healthy subjects	EEG	Participants sat calmly with michelia essential oil-infused scented or unscented filter paper on their noses.	Michelia leaf oil and pure linalool reduced alertness by decreasing beta wave and increasing fast alpha wave activity.
Hawiset (40)	2019	Thailand	80 healthy students aged between 18 and 22	Cognitive test Visual analog mood scales Blood pressure HR Salivary cortisol	G1: Coffee fragrance inhalation (5 min, 2 days) C: Placebo inhalation (5 min, 2 days)	Coffee fragrance inhalation enhanced cognitive functions (attention, memory quality, and speed) and increased mood alertness score.
Sona et al. (41)	2019	Germany	122 German students	Perceived Restorativeness Scale	Participants completed three cognitively demanding tasks within 50 min and then entered one of five resting conditions for 15 min.	Simulated natural or lounge settings were more pleasant and restorative, enhancing personal wellbeing (mood, fatigue, arousal). Adding a matching scent to an audiovisual simulation further improved resource recovery through increased scent pleasantness and enhanced feelings of fascination and transport.
Ohata et al. (42)	2020	Japan	26 healthy participants aged between 21 and 24	POMS Pupillary light reflex Fingertip temperature Flicker test Near-infrared spectroscopy measurement	G1: Inhaled Maillard vapor (2 min) G2: Inhaled 3DP vapor (2 min) G3: Inhaled DMHF vapor (2 min) C: Inhaled air (2 min)	Inhaling Maillard reaction and DMHF scents decreased anger-hostility and tension-anxiety moods, with DMHF also reducing fatigue-inertia mood. Both scents increased miosis rate and fingertip temperature, showing a relaxing effect through reduced flicker frequency and prefrontal cortex oxyhemoglobin levels. Inhaling 3DP or DMHF prompted parasympathetic dominance, lowering prefrontal cortex oxyhemoglobin levels by suppressing the sympathetic nervous system.

MFI, Multi-dimensional fatigue inventory; KSS, Karolinska Sleepiness Scale; STAI, Spielberger State-Trait Anxiety Inventory; HR, Heart rate; EEG, Electroencephalograph; VDT, Visual display terminal; VAS, Visual analog scale; POMS, Profile of Mood States; HRV, Heart rate variability; 3DP, 2,3-dimethylpyrazine; DMHF, 2,5-dimethyl-4-hydroxy-3 (2H)-furanone.

The utilization of scent to enhance alertness and alleviate fatigue has not only found application within the transportation industry but has also played a pivotal role in mitigating work-related exhaustion among healthcare professionals. In a 6-week experiment involving exercise groups, lavender groups, and control groups, the results demonstrated the positive impact and fatigue-reducing effect of concomitant exercise and the fragrance of lavender within the operating room for surgical technologists (25). In the study conducted by Nasiri and Boroomand, a cohort of 80 shift-working nurses was subjected to random assignment, with participants divided into either a control group or an intervention group (26). The intervention group was administered a solitary drop of rosemary essential oil onto their masks. The research revealed that rosemary had the ability to diminish drowsiness and enhance alertness among shift-working nurses.

The research encompasses not only subjects in good physical health but also individuals with specific health issues. Aromatherapy, as an adjunct therapy, has been employed to treat various ailments, and research suggests that scents can alleviate fatigue in patients. Mahdavikian et al. conducted a study where scented cotton balls were applied to the collars of cardiac patients every evening at 9 p.m. for 20 min over a period of seven consecutive days (3). The study found that aromatherapy with peppermint and lavender essential oils could alleviate fatigue in cardiac patients. Starting from the second day of hospitalization, inhalation of three drops of citrus aurantium essential oil or a placebo through absorbable patches connected to an oxygen mask for a duration of 20 min was administered consecutively for 2 days (27). The experimental results showed that citrus aurantium essential aroma could alleviate feelings of anxiety and fatigue among patients diagnosed with Acute Myocardial Infarction. Aromatherapy encompasses not only inhalation of essential oils but also includes aromatherapy massage. Hur et al. proposed that inhalation of fragrances coupled with self-abdominal massage might contribute to controlling blood glucose levels, reducing fatigue, and improving sleep quality in middle-aged women with prediabetic conditions (28). It pointed out that inhalation aromatherapy is effective in alleviating fatigue in hemodialysis patients, but aromatherapy massage has a stronger impact on fatigue compared to inhalation aromatherapy (29).

Research indicates that aromas have the potential to alleviate physical exhaustion experienced during exercise. Tianlong and Sim conducted a study to investigate the impacts of four recovery methods, including aromatherapy, massage, static rest, and acupoint acupressure, on the concentrations of fatigue substances and stress hormones after boxing training (30). The study found that aromatherapy with essential oils utilizing essential oils emerged as the most efficacious approach in ameliorating fatigue and diminishing levels of stress hormones. 20 university students participated in a 30-min treadmill running session in a room scented with orange fragrance (31). The research showed that the aroma reduced fatigue during exercise and had a positive impact on exercise satisfaction and adherence for the participants.

### 3.4 Measurement methods

Table 3 provides information on alertness measurement methods, including physiological signal detection, task performance evaluation, and questionnaire surveys. Physiological signal detection methods primarily involve techniques such as EEG (16, 18, 34), heart rate (18, 32), heart rate variability (HRV) (22, 23, 38), as well as eye movement characteristics (19, 21, 33), and others. Task performance evaluation includes measurements such as reaction time (32, 33) and task error rates (33). Questionnaires commonly employed include the Karolinska Sleepiness Scale (KSS) (18, 26, 35), Multi-dimensional Fatigue Inventory (MFI) (25, 27), and Profile of Mood States (POMS) (15, 37–39, 42), among others. Fatigue detection methods for driving also include vehicle driving characteristics, such as steering wheel features (21).

**Table 3:** Alertness measurement methods.

***N***.	**Measurement methods**		**No. of Included Studies**
1	Physiological signal	EEG	(16, 18, 24, 34)
		HR	(18, 32)
		HRV	(22, 23, 38)
		Eye Movement Characteristics	(19, 21, 33)
		Others	(23, 30, 42)
2	Questionnaire	KSS	(18, 24, 26, 35)
		MFI	(25, 27),
		POMS	(15, 37–39, 42)
		VAS	(36, 37)
		Others	(3, 20, 21, 23, 26, 28, 29, 31, 34, 36, 39–41)
3	Task performance		(22, 32, 33)
4	Vehicle driving characteristics		(21)

EEG, Electroencephalograph; HR, Heart rate; HRV, Heart rate variability; KSS, Karolinska Sleepiness Scale; MFI, Multi-dimensional fatigue inventory; POMS, Profile of Mood States; VAS, Visual analog scale.

### 3.5 Types of scent

Analysis of the aforementioned studies reveals that various aromas, including herbal plants, fruits, flowers, wood, and food, have an impact on fatigue, as summarized in Table 4.

**Table 4:** Types of scent.

**N**.	**Types of scent**		**No. of Included Studies**
1	Herb	Peppermint	(3, 18–24, 32, 33, 37)
		Rosemary	(19, 26, 30)
2	Citrus		(19, 27, 31, 34–37)
3	Flower	Lavender	(3, 25)
		Michelia	(16)
4	Wood		(15, 19, 38, 39)
5	Food		(40, 42)
6	Mixed fragrance		(28, 29, 41)

Regarding the scent of peppermint, research findings indicate that it can enhance driver alertness (18) and alleviate fatigue in patients with heart disease (3). Peppermint-flavored chewing gum has been found to improve exam performance, alertness, and attention, thereby enhancing cognitive abilities (32). In the scenario of prolonged performance of repetitive tasks, the aroma of peppermint directly enhances the recollection of information in instances of participant fatigue and mitigates the deterioration of alertness. Intermittent exposure to peppermint aroma has a slightly stronger enhancing effect on alertness and memory compared to continuous exposure (33). The scent of peppermint has been found to alleviate tension induced by block-building tasks and contribute to maintaining participants' vitality (37).

The aroma of rosemary has been found to enhance driver vigilance (19, 26), improve alertness in shift nurses (26), and alleviate exercise fatigue in boxing training (30).

Studies have shown that the aroma of citrus fruits has various effects: citrus aurantium essential oil can alleviate anxiety and fatigue in myocardial infarction patients (27); orange scent can reduce fatigue during exercise (31); the aroma of sweet orange can also alleviate fatigue after playing with blocks (37). The scent of lemon can improve alertness and prevent driver drowsiness (35). Shiikuwasha (Citrus depressa Hayata) essential oil has demonstrated the capacity to increase vigor and reduce fatigue (34). Japanese citrus fruits iyokan (Citrus iyo) and yuzu (Citrus junos) have been shown to reduce feelings of fatigue, enhance mental vitality, and improve task performance (36).

For floral fragrances, research has shown that the scent of lavender can alleviate fatigue in surgical technicians (25) and reduce fatigue in cardiac patients (3). On the other hand, inhalation of michelia essential oil has been found to decrease alertness (16).

For woody fragrances, studies have indicated that the scent of eucalyptus can alleviate fatigue while driving (19). The scents of Taiwania have been found to induce relaxation and suppress negative emotional states, including fatigue, confusion and depression. They also result in higher scores in terms of stimulation, determination, excitement, activity, uniqueness and intensity, but lower scores in terms of pleasure (15). Essential oil from fir has been shown to have physiological and psychological relaxation effects, significantly improving positive emotions related to vitality and reducing negative emotions related to tension-anxiety, depression, anger-hostility, and fatigue (38). Women tend to experience a greater sense of warmth and brightness in woody environments, while changes in indoor environments are more likely to make women feel confused and fatigued, while men exhibit a noticeable sense of vitality (39).

The aroma of food and beverages can impact alertness. A study by Hawiset investigated the impact of inhaling coffee aroma on the mood, memory and salivary cortisol levels of healthy young volunteers (40). The study found that a one-time inhalation of coffee aroma could enhance working memory and increase alertness. Aromas resulting from the glycine/glucose Maillard reaction have demonstrated the ability to elevate mood and induce physiological relaxation by inhibiting sympathetic activity (42).

The studies also involved combinations of multiple aromas. Adding congruent odors in audiovisual simulation indirectly facilitated emotional recovery, alleviated fatigue, and modulated arousal levels (41). Inhalation of lavender, cinnamon, geranium, grapefruit, neroli, and ylang-ylang in a ratio of 6:3:3:3:1:3 was found to alleviate fatigue in prediabetic women (28). Inhalation of lavender and sweet orange was shown to reduce fatigue in patients undergoing hemodialysis (29).

## 4 Discussion

### 4.1 Psychophysiological explanations

The sense of smell is primarily mediated by two distinct yet interconnected sensory pathways: the olfactory and somatosensory (trigeminal) systems (43).

The trigeminal chemosensory system enables the perception of sensations such as freshness, burning, stinging, or tickling from odorous stimuli (44–46). This system functions through the specific interaction of chemicals with trigeminal chemoreceptors located on the fibers of the trigeminal nerve, cranial nerve V. This process operates independently of olfactory processing, which involves the activation of the olfactory nerve, cranial nerve I (47).

The olfactory system comprises an intricate network consisting of a sensory organ (olfactory epithelium) as well as specific olfactory brain regions (olfactory bulb and higher olfactory cortex) (48). The interaction between the olfactory system and the brain involves the processes of odor perception, activation of olfactory receptors, neural signal transmission, olfactory nerve transmission, olfactory bulb transmission, and olfactory cortex processing (49–52). Sensory information from olfaction could additionally be directed to more advanced associative regions like the orbitofrontal cortex, thalamus, and hypothalamus. Research has found a close association between the neuroanatomy involved in olfaction and that related to emotional states, with several regions significantly overlapping in these two neuroanatomical circuits, such as the hippocampus, amygdala, and olfactory cortex itself (35). Therefore, through the sense of smell, it is possible to influence a person's emotions and states, and even enhance their alertness and alleviate fatigue.

Few scents produce purely olfactory or purely trigeminal sensations. The vast majority exhibit both odorant and irritant characteristics. The olfactory and trigeminal systems are closely linked and work in concert in the perception of odors (53).

Trigeminal odors directly influence sensory responses by stimulating the trigeminal nerve (the fifth cranial nerve), often accompanied by intense sensations. These odors are conveyed not only through the olfactory system but also via the trigeminal nerve, resulting in unique psychological and physiological reactions. Trigeminal odors can directly stimulate the trigeminal nerve, causing significant changes in physiological parameters such as heart rate and respiratory rate. For instance, capsaicin in chili peppers can induce physiological responses like heating and tearing through trigeminal stimulation, thereby enhancing arousal and attention (54). These odors typically evoke stronger sensations, such as tingling or burning, leading to more intense subjective experiences and emotional reactions through the activation of trigeminal nerve fibers. Studies have shown that menthol odor can improve alertness and cognitive performance while reducing fatigue (55), likely due to the cooling effect and strong sensory stimulation of menthol.

Non-trigeminal odors, such as floral and fruity scents, are primarily transmitted through the olfactory epithelium, activating the olfactory bulb and associated brain regions. These odors typically do not accompany sensations of irritation but influence emotional and cognitive responses through their aromatic properties. Non-trigeminal odors can modulate physiological parameters like heart rate and skin conductance by regulating nervous system activity. For instance, floral scents such as lavender have been shown to reduce stress, enhance relaxation, and improve sleep quality (56). These effects are mediated by the modulation of the autonomic nervous system and cortisol levels. Psychologically, non-trigeminal odors convey information via olfactory receptors, eliciting emotional and memory responses through the olfactory system.

Psychological hypotheses suggest that odor functions through affective learning, conscious perception, and belief/expectation. At the core of psychological hypotheses is the assertion that responses to odors are acquired through their association with emotional experiences, thereby imbuing odors with affective characteristics and exerting consistent effects on emotions, cognition, behavior, and physiology (57).

Odors exhibit high correlation and emotional arousal, consistent with the neuroanatomy of the limbic system. Odors can influence emotions (58). Pleasant odors can induce positive emotions, while unpleasant odors can evoke negative emotions (14).

Odors evoke memories associated with our past. Compared to signals sent to other sensory channels, much research has focused on the ability of odor signals to evoke autobiographical memories. Herz demonstrated that memories triggered by odors are considered to be more emotional compared to memories evoked by other types of stimuli (59). Thus, when we experience a particular odor and associate it with a specific emotional experience in our memory, it can evoke related emotions when encountered later (14).

Beliefs and expectations also have a significant impact on the outcomes of odor interventions. The results of a study by Campenni et al. showed that in the case of odorless placebos, physiological changes related to relaxation or stimulation could be induced solely through suggestion (60). Knasko also observed that suggesting the presence of a pleasant odor in a room (even when none was present) had positive effects on mood (61). These findings suggest that the chemical nature of the odor itself plays a secondary role in the emotional and subjective changes that occur in the presence of odor, and it is the meaning of the odor that triggers the subsequent psychological and/or physiological responses.

Overall, the research provides a physiological and psychological theoretical foundation for the application of scents in enhancing alertness, alleviating fatigue, and consequently reducing occupational health and safety hazards.

### 4.2 Scent intervention on fatigue driving

As a means to alleviate driving fatigue and reduce occupational health and safety hazards, the use of fragrance can effectively reduce the occurrence of traffic accidents, thereby improving road safety.

Research on the application of olfactory stimulation has also extended to the field of autonomous driving. In an effort to measure the influence of olfactory stimulation and takeover modality on takeover performance in conditional automated driving, Tang et al. observed that the peppermint odor as an auxiliary stimulation of auditory and tactile takeover requests improved takeover quality, as measured by Time to collision, longitudinal acceleration, lateral acceleration, and overshoot (22).

Scents have been found to exert an impact on the psychological state of drivers, and thereby affect their work performance. In a two-week experimental study involving 120 taxi drivers, it was observed that aromas can improve passenger mood and facilitate smoother driver-passenger communication. This had the effect of reducing driver tension, elevating their sense of pleasure, and ultimately boosting job satisfaction (62). Scent has the power to alter the driving behavior of individuals. Specifically, in a simulated driving experiment, the scent of peppermint was found to reduce the aggressive driving tendencies of participants (63).

Several studies have compared the effects of olfactory stimulation with visual and auditory stimulation on drivers. Bordegoni et al. conducted experiments to investigate whether olfactory stimulation could affect drivers' cognitive performance and found that olfactory stimulation had a better effect on improving drivers' attention than auditory stimulation (23). Additionally, during driving, olfactory stimulation had a masking effect on auditory stimulation (64). Dmitrenko et al. explored the application of peppermint, lavender and lemon scents in conveying three driving-related messages, and the study found that olfactory cues were less distracting than visual cues, allowing drivers to feel more comfortable and be more effective, while also making fewer errors (65). It appears that olfactory cues can affect the driver's psychological state, which in turn affects their work state.

### 4.3 Gender difference

Some studies have focused on either male or female participants, while others have included individuals of different genders. Research has shown that the impact of the same odor can vary between different genders (38). Shen et al. found that in a woody environment, women experienced fatigue while men felt more vitality (39). Studies have indicated that olfactory abilities differ based on gender (66, 67). Generally, women have a heightened sense of smell (68, 69), stronger scent identification skills (66), and greater olfactory potential compared to men (67). Oliveira-Pinto et al. discovered significant gender differences in the absolute number of cells in both neural and non-neural cells in the human olfactory bulb (70). Women had 43% more cells in the olfactory center of the brain and 50% more neurons, which are related to olfaction control. Therefore, due to physiological differences, the perception of odors can differ between genders, influencing the effects of odors on alertness enhancement. Gender factors should not be overlooked in studies investigating odor interventions for fatigue.

### 4.4 Measurement methods

Fatigue, as investigated in the studies, encompasses both physiological fatigue, such as exercise-induced fatigue and fatigue caused by work-related tasks, as well as psychological fatigue, often involving a combination of physiological and psychological aspects. Consequently, a comprehensive assessment of alertness is conducted through various methods, including physiological signal detection, task performance evaluation, and questionnaire surveys. Furthermore, many studies explore a combination of multiple indicators to assess alertness, rather than relying solely on a single fatigue indicator.

Fatigue is a complex psychological and physiological state, and the choice of fatigue identification methods should consider research and application scenarios, taking into account accuracy and low interference.

### 4.5 Scent intervention methods

#### 4.5.1 Scent presentation system

The effectiveness of scent intervention in fatigue management is closely related to the scent presentation system. The scent presentation system consists of two technical domains: scent generation and scent delivery.

Odor generation is determined by the use of odorants. Typically, odorants are in liquid form and are encapsulated, gelled, or impregnated into porous materials. Various methods can be employed to release odorant molecules based on their molecular forms, including natural evaporation, airflow-based volatilization, heating, airflow-based nebulization, direct nebulization-piezoelectric method, and ultrasonic nebulization.

Based on the location of odor-generation devices, there are two main types of scent presentation systems. One type is the fixed type, where scent generators are integrated into the surroundings. The other type is the portable type, where scent generators are attached to the body. The advantage of wearable devices is that the scent can be stably presented to the user. However, devices may interfere with occupational activities, potentially compromising safety.

Multiple aroma delivery technologies can be employed for fixed odor presentation systems, including natural diffusion, airflow, vortex rings, and tubes. Natural diffusion refers to the simple and uncontrolled spreading of scent in the air, such as with independent scent generators. Airflow refers to the transportation of scent carried by the air conditioning system. Vortex ring refers to the scent being carried by a vortex ring without diffusion, as the vortex maintains its shape. Funato et al. proposed that the vortex ring is the most suitable method for odor transmission and tested the stimulating effect of fragrance on drivers and its effective duration using the vortex ring method (21). Tubes refer to the scent being carried through a duct-like structure. These methods are suitable for installation in vehicles such as automobiles. Funato et al. conducted experiments to compare the aforementioned scent transmission techniques, and the results are presented in Table 5.

**Table 5:** Comparison of aroma delivery methodologies (21).

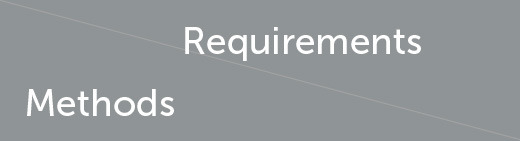	**No interference with driving operations**	**Installable as car accessory equipment**	**Prevention of reduced sensitivity due to the adaptation**
Natural diffusion	Good	Good	Poor
Airflow	Good	Good	Poor
Vortex ring	Good	Good	Good
Tube	Poor	Good	Good

Hirata conducted experiments and discovered the characteristic of olfactory fatigue, where the perceived intensity of odor rapidly increases upon odor release and then decreases due to fatigue (20). Based on the above analysis, it is evident that a fatigue intervention scent delivery system that does not interfere with occupational activities ensures safety, is easy to install and maintain, provides intermittent odor release, and is cost-effective, is the inevitable trend for the application of odor-based fatigue interventions in occupational environments.

#### 4.5.2 Scent intervention strategies

In the relevant studies, scent intervention strategies can be classified based on their intervention timing. There are pre-task interventions, where participants are exposed to scents for consecutive nights prior to the experiment (18). There are concurrent task interventions, where tasks are performed in an environment with scents present (31). There are also post-fatigue interventions, where drivers are subjected to scent interventions when experiencing fatigue (19). In terms of intervention duration, there are continuous exposure and intermittent exposure (19). Scent interventions can take the form of direct and indirect interventions. Direct interventions involve odor delivery through odor presentation systems. Indirect interventions include methods such as chewing flavored gum (32). Overall, considering the fatiguing nature of olfaction (20), intermittent scent release and low task interference scent intervention strategies are more suitable for practical applications.

Based on the reviewed studies, it is evident that peppermint fragrance has shown promising effects in alleviating fatigue. Moreover, there is an increasing trend in exploring the use of other fragrances such as citrus for enhancing alertness. Furthermore, fatigue alleviation measures are not limited to single scent interventions alone. Combining scents with other measures, such as combining scent with physical exercise, is also a research trend. Different strategies can work synergistically, with the positive effects of different strategies enhancing and even strengthening each other.

### 4.6 Limitations

One limitation of this review is that it did not include a search of non-English electronic databases, which may introduce a language bias (71). Considering the multiple influencing factors involved in scent intervention research, such as task variations, scent presentation techniques, environmental factors, and individual physiological differences among participants, there is currently no comprehensive evaluation system that can effectively consider these factors and measure the effectiveness of scent's impact on alertness in various studies.

## 5 Conclusions

Fatigue is a prevalent issue in contemporary society, affecting people's work efficiency and occupational health and safety. Research has shown that olfaction can significantly impact human alertness and fatigue through mechanisms including emotional regulation, neural influences, cognitive effects, and physiological responses. The scent, owing to its minimal interference, has garnered significant attention and is considered a promising avenue for mitigating fatigue. Our study aims to conduct a scoping review on the effects of olfactory interventions on human alertness while exploring their applications in different occupational settings. Our overarching goal is to bridge the existing gaps in the literature, ultimately offering valuable guidance for potential systematic reviews.

This review places particular emphasis on research within the domain of olfactory interventions for fatigue driving. It delves into various aspects, including participants' characteristics, measurement methods, and intervention methods. Most of the studies reviewed indicate that olfactory interventions have a positive impact on human alertness, demonstrating significant potential as an effective strategy for preventing occupational health hazards. This provides detailed and practical guidance for the actual application of olfactory interventions in alleviating fatigue and reducing occupational risks.

In order to provide detailed and practical guidance for the actual application of olfactory interventions in improving the occupational environment and reducing occupational risks, further research is needed to investigate the potential mechanisms, applications, and efficacy evaluation systems specific to fatigue olfactory interventions.

## Author contributions

XJ: Investigation, Methodology, Writing – original draft. KM: Project administration, Supervision, Writing – review & editing. XF: Data curation, Visualization, Writing – review & editing.
